# Pain in axial spondyloarthritis: role of the JAK/STAT pathway

**DOI:** 10.3389/fimmu.2024.1341981

**Published:** 2024-02-23

**Authors:** Carlo Selmi, Maria Sole Chimenti, Lucia Novelli, Bhumik K. Parikh, Francesca Morello, Kurt de Vlam, Francesco Ciccia

**Affiliations:** ^1^ Department of Rheumatology and Clinical Immunology, IRCCS Humanitas Research Hospital, Milan, Italy; ^2^ Department of Biomedical Sciences, Humanitas University, Milan, Italy; ^3^ Rheumatology, Allergology and Clinical Immunology, Department of “Medicina dei Sistemi”, University of Rome Tor Vergata, Rome, Italy; ^4^ Medical Department, AbbVie srl, Rome, Italy; ^5^ Global Medical Affairs, AbbVie, Inc., Mettawa, IL, United States; ^6^ Department of Rheumatology, University Hospital Leuven, Leuven, Belgium; ^7^ Skeletal Biology and Engineering Research Center (SBE), Department of Development and Regeneration, KULeuven, Leuven, Belgium; ^8^ Department of Precision Medicine Napoli, Università degli Studi della Campania “Luigi Vanvitelli”, Caserta, Italy

**Keywords:** JAK/STAT signaling pathway, small molecule inhibitor, axial spondyloarthritis, pain, residual disease

## Abstract

Axial spondyloarthritis (axSpA) is a chronic inflammatory disease that is characterized by new bone formation in the axial musculoskeletal system, with X-ray discriminating between radiographic and non-radiographic forms. Current therapeutic options include non-steroidal anti-inflammatory drugs in addition to biological disease-modifying anti-rheumatic drugs that specifically target tumor necrosis factor-alpha (TNFα) or interleukin (IL)-17. Pain is the most critical symptom for axSpA patients, significantly contributing to the burden of disease and impacting daily life. While the inflammatory process exerts a major role in determining pain in the early phases of the disease, the symptom may also result from mechanical and neuromuscular causes that require complex, multi-faceted pharmacologic and non-pharmacologic treatment, especially in the later phases. In clinical practice, pain often persists and does not respond further despite the absence of inflammatory disease activity. Cytokines involved in axSpA pathogenesis interact directly/indirectly with the Janus kinase (JAK)/signal transducer and activator of transcription (STAT) signaling cascade, a fundamental component in the origin and development of spondyloarthropathies. The JAK/STAT pathway also plays an important role in nociception, and new-generation JAK inhibitors have demonstrated rapid pain relief. We provide a comprehensive review of the different pain types observed in axSpA and the potential role of JAK/STAT signaling in this context, with specific focus on data from preclinical studies and data from clinical trials with JAK inhibitors.

## Introduction

Spondyloarthropathies involve a cluster of chronic inflammatory diseases, including psoriatic arthritis (PsA) and axial spondyloarthritis (axSpA), in addition to less frequent forms such as enteropathic arthritis or reactive arthritis (1). AxSpA is a chronic disease that is characterized by inflammation and the formation of new bone of the axial skeleton, particularly localized to the sacroiliac joints and spine and includes the two subtypes, non-radiographic axSpA (nr-axSpA) and radiographic axSpA (r-axSpA; classically known as ankylosing spondylitis) (2–4), depending on findings at X-ray imaging.

Treatment options currently available for patients with axSpA include non-steroidal anti-inflammatory drugs (NSAIDs) and biologic disease-modifying anti-rheumatic drugs (bDMARDs) that target tumor necrosis factor-alpha (TNFα) or interleukin (IL)-17 in addition to targeted synthetic DMARDs (tsDMARDs) that target the Janus kinase (JAK) and signal transducer and activator of transcription (STAT) pathway (1, 5–12).

The efficacy of inhibitors targeting TNFα and IL17 in improving signs and symptoms of axSpA has been documented in randomized controlled trials (RCTs) (1, 5, 6), but observational studies and real-life evidence demonstrate that a significant proportion of patients still do not achieve low disease activity (LDA) or remission status, tend to lose response after time, or are not candidates for these treatments (13, 14), particularly for the concomitant presence of comorbidities or associated conditions (15).

JAK/STATs act as key transmitters in both pro-inflammatory and anti-inflammatory signals in immunoregulation (16–18), and pathogenic pathways in axSpA are directly and indirectly mediated by JAK-dependent cytokines (19), thereby supporting a role for JAK inhibitors as a therapeutic choice in axSpA with JAK inhibition offering a favorable and potentially more comprehensive approach, by blocking several cytokines simultaneously (17, 20).

Chronic pain, particularly inflammatory back pain, is a frequently occurring symptom reported in patients with axSpA, and treatments focused on the reduction of pain are of major clinical relevance (21–23), as this symptom is associated with lower quality of life (QoL), fatigue, functional and work productivity impairment (24–26).

Preclinical data from several studies in the literature emphasize the role of the JAK/STAT signaling pathway in nociception; for instance, evidence from *in vivo* neuropathic pain models shows that the JAK/STAT3 pathway may regulate spinal astrocyte proliferation and maintenance of neuropathic pain in rodents (27). Moreover, data from recent trials using JAK inhibitors have demonstrated rapid and sustained pain relief (28–30).

JAK inhibitors have therefore gained increasing attention among rheumatologists for their possible use in the management of axSpA.

Prior to discussing the role of JAK/STAT in pain, we should note that differences do exist between different JAK inhibitors, including the chemical structure, inhibition potency, metabolism, and urinary excretion profile (18, 20, 31, 32). Consistent with the mechanism of action of different JAK inhibitors, *in vivo* studies have shown that tofacitinib preferentially inhibits both JAK1 and JAK3 and partially inhibits JAK2. Filgotinib is a selective JAK1 inhibitor, and evidence indicates that it can reduce levels of circulating proinflammatory cytokines as well as chemokines, adhesion molecules, and matrix remodeling markers associated with axSpA (33). Upadacitinib exerts direct inhibitory activity on several JAK1-dependent factors (IFNα/β, IFNγ, IL2, IL5, IL6, and IL7) and indirectly on several JAK1-independent pathways (TNFα, IL1, IL17, IL18, and IL23) (34), leading to the inhibition of cytokine-triggered events, such as leukocyte activation and migration, inflammation, and damage to connective tissue.

In this narrative review, we discuss pain in axSpA, the role of the JAK/STAT signaling pathway in nociception, and the results from recent clinical trials evaluating the use of currently approved JAK inhibitors in axSpA.

## Burden of disease and residual disease in axSpA

While considered in remission or LDA, a high proportion of patients with SpA continue to manifest a significant reduction in their QoL (35–40). Patients with axSpA frequently have an elevated burden of disease with a significant reduction in their QoL attributed to chronic inflammation that leads to chronic pain, joint stiffness, structural damage, and reduced function in addition to fatigue (41, 42). AxSpA has a pronounced impact on patients’ daily lives, and several studies have shown that impaired functional disability is associated with axSpA (41, 43–45). In addition, the detrimental impact of axSpA on mental health (e.g., anxiety and depression) is also well documented (46–48). In fact, coexisting conditions (i.e., cardiovascular disease and anxiety/depression) may further impact the QoL and physical function (49, 50).

To date, there are only a few studies that have specifically explored the residual burden of disease in patients with axSpA (38–40). A cross-sectional study performed across 23 rheumatology centers in Italy included 480 adults with axSpA classified according to the Assessment in SpondyloArthritis International Society (ASAS) criteria and evaluated how residual disease impacts patients’ QoL (39). Although classified with inactive disease after advanced therapy, approximately 50% of patients had mild pain/discomfort, and ∼4% reported moderate pain/discomfort according to the EuroQoL 5-Dimension 5-Level (EQ-5D-5L) questionnaire. Among axSpA patients in clinical remission/LDA, ≥25% of patients in remission/LDA status were still burdened by residual disease, which was mainly characterized by pain and fatigue (39). Similar findings were also observed in a study from Singapore (40), as one-third of the 262 patients with axSpA who achieved LDA were burdened with residual disease in musculoskeletal manifestations, including pain and fatigue. In a study undertaken in the Netherlands that included 267 patients with LDA, the proportion of patients burdened by residual disease was 42.7% (38). Furthermore, multivariate regression analysis revealed that fatigue occurred with greater severity and frequency in female patients (38).

Indeed, gender differences exist with regard to the diagnostic journey and management of disease, including variations in healthcare that may favor the earlier detection and diagnosis of axSpA in men (51–55). While men with axSpA have a worse radiologic prognosis, women have a higher burden of disease and report higher levels of pain, especially in longer existing axSpA and neuropathic and widespread pain (56) that is associated with diagnostic delay and less responsiveness to TNF inhibitor (TNFi) treatment (54, 55, 57). Similar results have also been observed for the IL17 inhibitor secukinumab in the PREVENT study where male patients showed higher relative responses compared to female patients (58).

## Pain in axSpA

**Figure 1:**
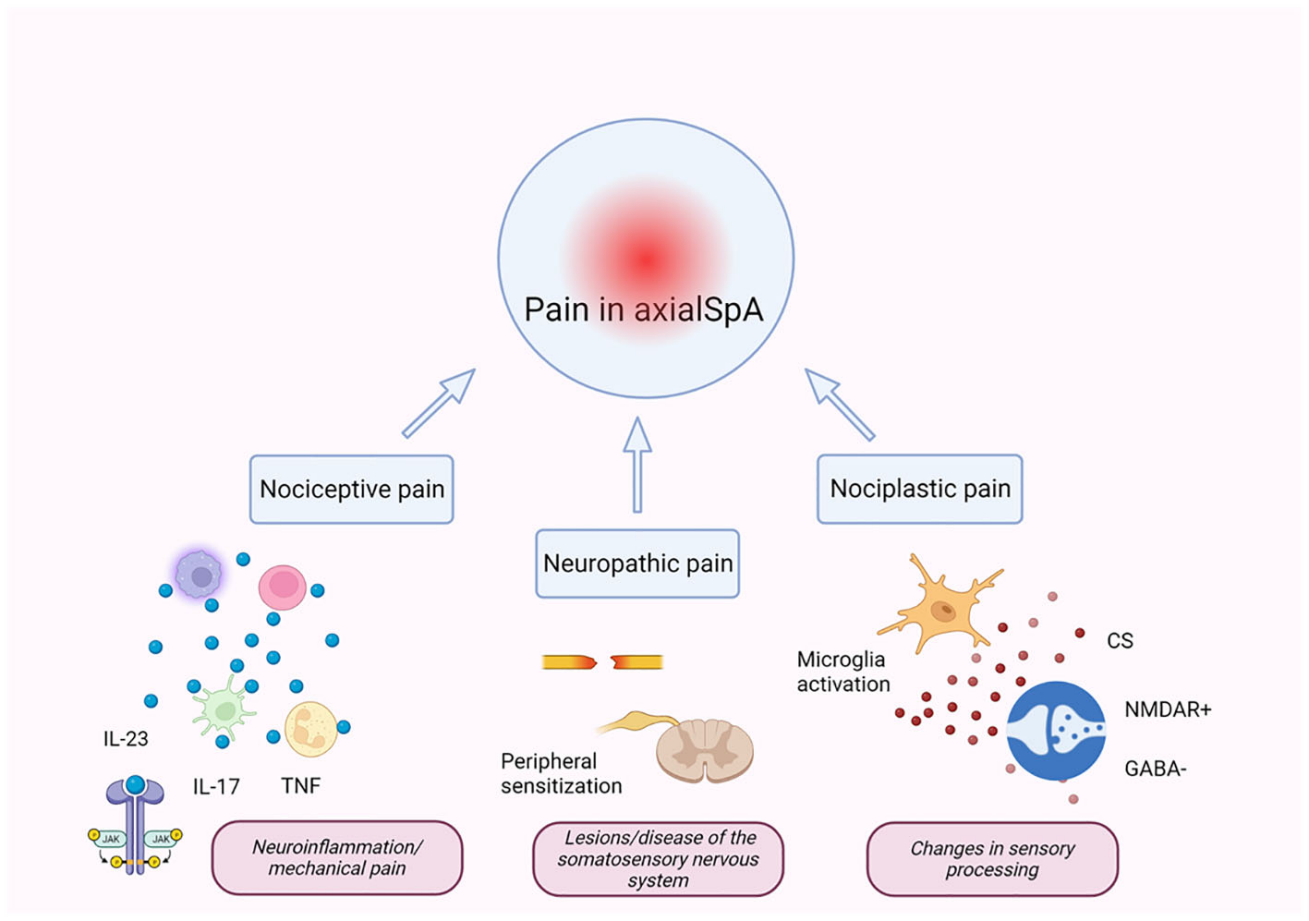
Mechanisms of pain in axSpA. Pain in axSpA is the result of the combination of three main types of pain: nociceptive, neuropathic, and nociplastic (66–68). Chronic inflammation in axSpA is driven by several pro-inflammatory cytokines (directly or indirectly mediated by the JAK/STAT pathway), which act on nociceptors, lowering the activation thresholds of transducers for evoked stimuli leading to increased pain and peripheral sensitization. Peripheral inflammatory mediators can alter pain-processing regions of the brain, leading to sensory hypersensitivity and central sensitization. Central sensitization is the main pathophysiological mechanism for developing nociplastic pain and is due to different mechanisms including over-activated glial cell-derived signals, potentiation of excitatory signaling of *N*-methyl-d-aspartate (NMDA) receptors, and decrease in the inhibitory neurotransmitter gamma-aminobutyric acid (GABA). IL, interleukin; TNF, tumor necrosis factor; CS, central sensitization; NMDAR, *N*-methyl-d-aspartate receptor; axSpA, axial spondyloarthritis. The figure was created using BioRender.com.

As in the case of the majority of rheumatic diseases, pain is recognized as an important and frequently occurring symptom of axSpA, which can fluctuate and can be persistent (59–62). Pain in axSpA is the result of variable combinations of three main types, i.e., nociceptive pain, due to inflammation or mechanical structural alterations; neuropathic pain, due to nerve damage or disease of the somatosensory nervous system; and nociplastic pain, caused by altered mechanisms of pain (63–68) (**Figure 1**). Nociception is defined as the process by which thermal, mechanical, or chemical stimuli stimulate nociceptors in nociceptive sensory neurons (69). Nociceptive sensory neuron cell bodies are primarily located in the dorsal root ganglia and have a peripheral axonal branch that innervates their target organ in addition to a central branch that innervates the spinal cord. Nociceptor activation only occurs when the level of intensity of the stimulus applied reaches a range of danger and damage (69). The current recognized definition of neuropathic pain, according to the International Association for the Study of Pain, is “pain induced by a lesion or disease of the somatosensory nervous system” (70). In chronic inflammatory conditions, periodic pain often leads to chronic pain, comprised of chronic widespread pain (CWP) and chronic localized pain (71). Central and peripheral sensitization maintains the continuation of chronic pain and is recognized as an atypical mechanism that controls pain (72). Synaptic plasticity in central sensitization is a condition that is characterized by an increase in neuronal responsiveness in central pain pathways in response to painful stimuli. This is regarded as a significant non-nociceptive pain mechanism that is derived from altered processing of central nervous system pain and can occur in the absence of peripheral injury or concomitant inflammation (73). However, inflammation can play the role of a trigger, and the neuroinflammatory process is recognized to contribute to central sensitization (74). Recently, awareness and concern with regard to treatment and classification difficulties in patients with axSpA and concomitant CWP have increased, mainly due to inadequate response to anti-rheumatic drugs and concomitant fibromyalgia (75–77).

Despite the significant impact that CWP has in axSpA, there are actually few studies available in this area. In r-axSpA, concomitant fibromyalgia has been shown to occur in patients with a prevalence of 4%–15% (78, 79) and higher for nr-axSpA (24%) (80). When not limited to the presence of fibromyalgia, concomitant CWP has been shown to occur in approximately 50% of r-axSpA cases (62). A greater number of pain regions and higher intensity of pain have been shown to emerge as important risk factors for CWP (81). The location and spread of CWP are different in men and women and are associated with a worsening of clinical status (82).

Although differences are recognized to exist between r-axSpA and nr-axSpA (83), few studies have evaluated differences in these subtypes with regard to CWP. In the SPARTAKUS cohort performed in Sweden (84), 43% of whom had CWP, the r-axSpA group tended to be older, had a higher frequency of male gender, had a longer history of symptoms and poorer spinal mobility, and had a twofold likelihood of being smokers compared to patients with nr-axSpA. However, the sensitivity, intensity, threshold, tolerance of pain, and temporal summation index were all similar across r-axSpA and nr-axSpA groups.

In rheumatic patients, residual chronic pain is still an unmet need despite the achievement of optimal control of the inflammatory disease. Neurogenic-mediated inflammation is inflammation based on the stimulation of nociceptive pathways. The development of chronic pain may be due to other processes beyond inflammation or structural damage such as psychological and environmental factors. The residual pain can be either unrecognized neuropathic pain (damage to neurons or central nervous system (CNS)) or nociplastic pain (including fibromyalgia and sensitization). There are arguments that sensitization might be dependent on the presence of some cytokines such as IL17, GM-CSF, and IL6 (85, 86). The extent of the burden or problem of residual pain has been heavily debated in other rheumatic diseases beyond axSpA, such as rheumatoid arthritis (85). Moreover, the concomitant presence of fibromyalgia syndrome (FMS), also an important comorbidity seen in axSpA (87), should be carefully considered. Indeed, it is important that nociplastic and neuropathic pain must be distinguished from residual inflammatory pain (85). In a multicenter, cross-sectional, observational study involving psoriatic patients, the association between patient-acceptable symptom state (PASS) and disease activity index for psoriatic arthritis (DAPSA) could be biased due to the presence of FMS (88). Indeed, the concomitant presence of FMS, which contributes to chronic residual pain, can influence the patient’s perception of the disease (88). In another multicenter observational study, the negative impact of pain catastrophizing on disease activity in patients with psoriatic arthritis (N = 135) and axSpA (N = 71) was evaluated. It was observed that a high level of the Pain Catastrophizing Scale was associated with a high level of disease activity (89). This study suggests that many psychometric variables that are independent of the inflammatory process are able to influence patients’ perception of the disease (and related patient outcome measures), significantly impacting the achievement of remission or LDA in inflammatory arthritis (89). Further studies (e.g., mediation analysis) are needed to confirm this hypothesis.

Information is still currently lacking with regard to mechanisms of pain that are not related to inflammation and whether neuropathic pain may be related to inflammation in axSpA (90–92).

Only through gaining an improved understanding of the various types of pain in different axSpA patients and improving the design of tools and assessments for the detection and measurement of pain can the appropriate treatment for pain management be achieved.

## The JAK/STAT signaling pathway in pain

JAK/STAT signaling has been shown to play a key role in the production of both pro-nociceptive and anti-nociceptive cytokines (93, 94), thus resulting in the regulation of nociception (95). More specifically, evidence from experimental models of pain shows that alterations to the JAK/STAT signaling pathway are associated with the modulation of pain. The majority of these studies were also based on models of neuropathic pain. Dominguez and colleagues demonstrated that lesions to the spinal nerve result in the rapid activation of JAK/STAT3 in the dorsal spinal cord microglia together with increased levels of spinal IL6 (96). JAK/STAT3 inactivation in rodent dorsal spinal cord glia through local, lentiviral-mediated production of the suppressor of cytokine signaling-3 prevented the abnormal expression of IL6, CCL2, and ATF3 induced in the spinal cord with marked attenuation of mechanical allodynia (96). In a different murine model, it was demonstrated that nerve injury-induced astrocyte proliferation requires activation of the JAK/STAT3 signaling pathway (27). The authors observed the STAT3 nuclear translocation in dorsal horn astrocytes following nerve injury and JAK inhibition in rats with nerve injury was shown to decrease the number of proliferating dorsal horn astrocytes and recovery from tactile allodynia, a recognized sign of neuropathic pain (27). It is also recognized that the activation of the RAGE/STAT3 pathway occurs during central spinal sensitization and lumbar disc pain (97, 98).

Alterations to the JAK/STAT3 pathways have been documented in other models of pain in rats (e.g., electroacupuncture and oxaliplatin), where an increase in STAT3 was observed (99–101) or the collagen-induced arthritis in mice in which baicalin, an anti-inflammatory agent, decreased pain as well as concomitant suppression of JAK/STAT3 signaling (102).

A recent study undertaken in Hungary is worth mentioning since it also documents the importance of the JAK/STAT pathway in the context of pain. Nociceptive pain in complex regional pain syndrome can be the result of persistent inflammation (103), which shares some similarities and bears relevance to axSpA. Using a complex regional pain syndrome mouse model by transcriptomic analyses, Pohóczky and colleagues evaluated TNF and JAK/STAT pathways as possible novel targets (104). Unbiased transcriptomic analysis of the dorsal root ganglia was performed in a passive transfer-trauma mouse model, and the predicted pathways were confirmed by pharmacological analysis. Pathway analysis highlighted the involvement of TNF and JAK/STAT signaling since treatment with the TNF inhibitor etanercept or JAK inhibitor tofacitinib reduced microglia and astrocyte markers in pain-associated central nervous system regions (104). This study further underlines the relevance of the JAK/STAT pathway in pain.

Although the contribution of the JAK/STAT pathway to nociception still remains to be further clarified, it is recognized that several cytokine receptors, such as IL6R, IL1R, IL10R, and interferon (IFN)-γR, are expressed on afferent nociceptors, and there is evidence that cytokines acting at these receptors are associated with the modulation of pain (105).

It is important to note that activation of the JAK/STAT signaling pathway can reduce or intensify the level of pain, depending on the activation of specific intracellular mechanisms (93). In this regard, while the anti-nociceptive cytokine, IL10, and the pro-nociceptive cytokine, IL6, can activate the JAK1/STAT3 pathway, differences in downstream signaling can occur, resulting in anti-nociceptive or pro-nociceptive transmission, respectively (93, 106, 107).

Collectively, results from these experimental studies support a direct role of the JAK/STAT signaling pathway in pain nociception. Data on JAK/STAT pathway involvement in nociplastic pain are lacking; however, considering the role of the JAK/STAT and their dependent and independent cytokines in central sensitization, it is possible to speculate on a positive effect of JAK inhibitors on central chronic pain, although additional studies are warranted to demonstrate this hypothesis.

## Efficacy of JAK inhibitors in axSpA

To date, clinical trials have evaluated three different JAK inhibitors (tofacitinib, filgotinib, and upadacitinib) in axSpA (108–114), and tofacitinib and upadacitinib are currently approved for the treatment of r-axSpA and both r-axSpA and nr-axSpA, respectively.

In all trials, the efficacy and safety of JAK inhibitors compared to placebo control was evaluated in axSpA patients with an inadequate response/intolerance to NSAIDs (NSAIDs-IR) with or without a prior inadequate response to bDMARDS (bDMARDs-IR). All seven trials achieved the primary endpoints in addition to the main secondary endpoints (108–113).

Tofacitinib was evaluated in a phase 3 trial including 269 patients with r-axSpA and NSAIDs-IR (109). For the primary outcome, a higher ASAS20 response was observed at Week 16 in the tofacitinib group compared to the placebo group (56.4% compared to 29.4%; p < 0.001).

The efficacy and safety of filgotinib (115) (not currently approved for the treatment of patients with axSpA) were evaluated in 161 patients with axSpA in TORTUGA, a double-blind, placebo-controlled phase 2 trial (110). Patients were treated with NSAIDs-IR or anti-TNF agents, and Ankylosing Spondylitis Disease Activity Score (ASDAS) score at Week 12 improved in the filgotinib group compared to the placebo group (Δ of −1.47 ± 1.04 compared to −0.57 ± 0.82, respectively; p < 0.0001). The mean difference from baseline after 12 weeks in levels of high-sensitivity C-reactive protein (CRP) was also significantly lower in the filgotinib group (−10.8 ± 13.9 mg/L) compared to the placebo control (−2.2 ± 17.4 mg/L; p < 0.0001) (110).

Upadacitinib has been evaluated in a phase 2/3 double-blind, placebo-controlled RCT (SELECT-AXIS-1) in patients with r-axSpA with an NSAIDs-IR. Moreover, upadacitinib has been evaluated in SELECT-AXIS-2, a phase 3 study program, which was conducted under a master protocol including two distinct studies: Study 1 enrolled r-axSpA patients with bDMARDs-IR, and Study 2 enrolled nr-axSpA patients with an NSAIDs-IR who were either bDMARD-naïve or bDMARDs-IR (111, 112, 114).

In SELECT-AXIS-1, the primary endpoint of the trial was achieved; ASAS40 response at Week 14 was significantly greater in the upadacitinib group compared to the placebo group (52% compared to 26%; p = 0.0003).In Study 1 of SELECT-AXIS-2, a significantly higher number of patients achieved ASAS40 response at Week 14 (primary endpoint) with upadacitinib compared to placebo (45% compared to 18%; p < 0.0001).

In the SELECT-AXIS-2 Study 2, significantly more patients treated with upadacitinib achieved ASAS40 at Week 14 compared to placebo (45% compared to 23%; p < 0.0001).

## Pain reduction and other patient-reported outcomes in JAK inhibitor clinical trials

Several *post-hoc* analyses of phase 3 trials and other pooled analyses have specifically investigated the effect of tofacitinib and upadacitinib on pain reduction in axSpA (28–30, 116, 117), as patient-reported outcomes were not primary endpoints in these trials. No *post-hoc* or sub-analysis of the phase 2 TORTUGA trial has specifically evaluated pain as an outcome, although these patient-reported measures may be assessed following the conclusion of the ongoing phase 3 OLINGUITO trial (NCT05785611) (118).

In the evaluation of tofacitinib, Ogdie and colleagues collected data from patients with r-axSpA in seven tofacitinib studies on 3,330 patients (117) and reported an improvement in pain compared to placebo by Week 12. Specifically, the change from baseline in SF-36v2 bodily pain domain score improved significantly in tofacitinib-treated patients compared to the placebo group at Week 12, and the proportion of patients who answered “yes” to ankylosing spondylitis QoL (ASQoL) Question 9 (“I have unbearable pain”) or Question 14 (“The pain is always there”) decreased from baseline to Week 12.

Kristensen evaluated the association between fatigue, back pain, morning stiffness, and tofacitinib treatment in patients with r-axSpA, using mediation modeling (116) on the pooled data from two trials (370 patients). This analysis revealed that the major effect (~84%) of tofacitinib on fatigue is reducing morning stiffness. In a *post-hoc* analysis of the phase 3 trial including 269 patients with r-axSpA and NSAIDs-IR treated with tofacitinib *vs.* placebo (109), Navarro-Compán and colleagues specifically evaluated the effect of tofacitinib on pain, fatigue, health-related quality of life, and work productivity (29). After 16 weeks, patients in the tofacitinib arm showed an improvement in Bath Ankylosing Spondylitis Disease Activity Index (BASDAI) overall spinal pain (mean least squares difference, −2.85 *vs.* −1.34), BASDAI fatigue (−2.36 *vs.* −1.08), ASQoL (−4.03 *vs.* −2.01), and work productivity and activity impairment overall work impairment (−21.49 *vs.* −7.64) (all p < 0.001). The improvement in these outcome measures continued up to Week 48.

McInnes and colleagues evaluated the effect of upadacitinib on pain outcomes in patients with active PsA or axSpA across three RCTs (SELECT-PsA 1 and 2 for PsA; SELECT-AXIS 1 for axSpA) (28). Significant improvements in pain outcomes across different endpoints with upadacitinib were consistently observed over 1 year in patients with active PsA or r-axSpA who had either bDMARDs-IR (PsA studies) or were biologic-naïve with NSAIDs-IR (r-axSpA study). Similar to results observed in PsA, in SELECT-AXIS-1, a higher proportion of upadacitinib-treated patients with r-axSpA showed clinically relevant improvement for the assessment of global pain compared to placebo. The responses achieved at Week 2 increased with time and were maintained up to 64 weeks with ≥30% and ≥50% reduction in the patient global assessment of pain and minimal clinically important difference (MCID) and much better improvement in pain (achieved by 72% to 83% of patients), and 54% of patients achieved ≥70% decrease in pain at Week 64. In addition, the mean change from baseline in patient assessment of back pain, BASDAI question 2, and nocturnal back pain was significantly greater for upadacitinib compared to placebo as early as Week 2 and was consistently maintained at each time point through Week 14 (28). A higher proportion of patients treated with upadacitinib also achieved pain ≤1 numeric rating scale and ≤2 numeric rating scale compared to placebo up to Week 14 and patients who switched from placebo to upadacitinib treatment achieved a similar reduction in level of pain (28).

A *post-hoc* analysis of SELECT-AXIS 1 evaluated the association between clinically meaningful back pain improvement and patient−reported outcomes and disease activity (119). A significantly greater proportion of patients with AS achieved meaningful improvement in back pain with upadacitinib *vs.* placebo starting at Week 2. Improvement in back pain continued over time with over 70% of patients on upadacitinib reaching a meaningful improvement at Week 52. This *post-hoc* analysis showed that meaningful back pain improvement was associated with consistent and clinically meaningful improvement in other patient-reported outcomes and achievement of important measures of AS disease activity.

The effect of upadacitinib on total back pain and nocturnal back pain was also evaluated in the SELECT-AXIS 2 program (Study 1 and Study 2) within the multiplicity-controlled secondary endpoint hierarchy (114). In both studies, the average change from baseline to Week 14 in total back pain and nocturnal back pain was significantly greater in patients treated with upadacitinib compared to the placebo group (p < 0.001 for both comparisons). Furthermore, a *post-hoc* analysis of the SELECT-AXIS 2 Study 1 was conducted by Baraliakos and colleagues to further assess the efficacy of upadacitinib on several pain assessments in r-axSpA patients with bDMARDs-IR (120). Higher proportions of upadacitinib-treated patients achieved rapid and clinically relevant improvement in pain compared to placebo-treated patients by Week 2 that were maintained through Week 14 across a range of pain assessments, including ≥30%, ≥50%, and ≥70% reductions in patient global assessment of pain, total back pain, and nocturnal back pain. Similar trends were observed for the proportion of patients achieving MCID and much better improvement across the pain outcomes.

A *post-hoc* analysis of SELECT-AXIS 2 evaluated the effect of upadacitinib *vs.* placebo on health-related quality of life and work productivity in patients with active nr-axSpA (121). After 14 weeks, a higher proportion of patients treated with 15 mg upadacitinib reported clinically meaningful improvements ≥MCID *vs.* placebo in the patient-reported outcome measures ASQoL (62.6 *vs.* 40.9%; p ≤ 0.001), ASAS Health Index (44.8 *vs.* 28.8%; p ≤ 0.01), and Short Form-36 Physical Component Summary (69.3 *vs.* 52.0%; p ≤ 0.01).

Overall, data emerging from *post-hoc* analyses of RCTs point toward an important reduction in pain measures, frequently associated with a similar temporal reduction in patient-related outcome measures.

While these data suggest that JAK inhibitors are effective in managing nociceptive pain, there are limited clinical data and knowledge on the effect of JAK inhibitors on other types of pain, including neuropathic and nociplastic pain. The UPSTAND study (NCT04846244), an ongoing multi-country real-world observational study, is evaluating the effectiveness of upadacitinib on different pain types for up to 12 months in patients with r-axSpA (122). The primary outcome measures in the UPSTAND study are the proportion of patients with a total spinal pain score <4 with ≥2-unit improvement from baseline at Week 12 and the proportion of Week 12 responders who maintained this level of improvement at Week 52. The effect of upadacitinib on neuropathic pain and nociplastic pain is being assessed using the painDETECT questionnaire and the Widespread Pain Index/Symptom Severity Scale scores, respectively. Once available, data from this study will provide the first evidence of the impact of JAK inhibition on different types of pain in axSpA.

## Conclusions

There is a rising awareness regarding the burden of pain in axSpA and our current understanding of the JAK/STAT pathway in nociception, based on preclinical studies and phase III trials. Data from experimental studies have shown that the JAK/STAT signaling pathway is involved in the production of pronociceptive and pro-inflammatory cytokines, suggesting that this signaling pathway may be involved in the regulation of nociception. Although in remission or LDA, many patients with axSpA are still burdened with residual disease, and approximately half of patients with inactive disease according to ASDAS criteria have mild pain/discomfort, and up to one-quarter of patients in remission are still burdened by pain and fatigue. Moving from findings from preclinical studies, results from phase 3 RCTs demonstrate that JAK inhibitors such as tofacitinib and upadacitinib can improve disease severity in axSpA patients. Several *post-hoc* analyses of phase 3 efficacy trials and other studies have specifically investigated the important clinical benefit afforded by different JAK inhibitors on reducing pain in axSpA in both the short and long term. Taken together, the use of JAK inhibitors holds promise in the management of patients with axSpA, particularly the subset of patients still burdened with residual disease and pain. Further studies evaluating the effect of this class of therapies on non-nociceptive pain types are needed to understand the various pain mechanisms in axSpA and their relevance in predicting treatment response.

## Literature search

PubMed/Medline (until May 2023) was searched using the following keywords: [“axial spondyloarthritis” or “axial spa”] AND [pain or nociceptic or nociplastic or neuropathic). A second search was performed to identify studies examining JAK/STAT in axial spondyloarthritis. Studies that were not published in the English language in addition to hand-selected case studies, abstracts, letters, and reviews were excluded. Articles not related or not relevant to pain in axSpA or the topic discussed were also removed.

## Author contributions

CS: Conceptualization, Visualization, Writing – original draft, Writing – review & editing. MC: Conceptualization, Visualization, Writing – original draft, Writing – review & editing. LN: Conceptualization, Supervision, Visualization, Writing – original draft, Writing – review & editing. BP: Conceptualization, Visualization, Writing – original draft, Writing – review & editing. FM: Conceptualization, Visualization, Writing – original draft, Writing – review & editing. KV: Conceptualization, Supervision, Visualization, Writing – original draft, Writing – review & editing. FC: Conceptualization, Visualization, Writing – original draft, Writing – review & editing.
